# Diagnostic Challenges in Acute Basilar Artery Occlusion Successfully Treated With Mechanical Thrombectomy: A Case Report

**DOI:** 10.7759/cureus.105332

**Published:** 2026-03-16

**Authors:** Alexis A Palacios, Jose W Valverde, Jose C Serrano Reyes, Rolando L Jaen, Jose R Valdes

**Affiliations:** 1 Interventional Neuroradiology, Hospiten Paitilla, Panama City, PAN; 2 Critical Care Medicine, Hospiten Paitilla, Panama City, PAN; 3 General Medicine, Hospiten Paitilla, Panama City, PAN; 4 Internal Medicine, Hospiten Paitilla, Panama City, PAN; 5 Emergency Medicine, Hospiten Paitilla, Panama City, PAN

**Keywords:** basilar artery occlusion (bao), endovascular treatment (evt), ischemic stroke (is), mechanical thrombectomy (mt), posterior circulation stroke

## Abstract

Posterior circulation strokes are uncommon but are associated with high morbidity and mortality. We report the case of an 80-year-old woman with untreated hypertension who presented with sudden-onset vertigo, vomiting, gait instability, dysmetria, altered mental status, and oculomotor abnormalities without clear focal motor deficits. Initial noncontrast brain computed tomography showed no acute hemorrhage, while CT angiography demonstrated the absence of contrast opacification of the basilar artery, consistent with acute basilar artery occlusion (BAO). Baseline imaging suggested limited established infarction. The patient underwent urgent digital subtraction angiography followed by mechanical thrombectomy via the left vertebral artery, achieving complete recanalization. The time from symptom onset to successful reperfusion was nine hours. Neurological recovery was rapid, with full return to baseline functional status within 24 hours (modified Rankin Scale (mRS) score: 0). Follow-up brain magnetic resonance imaging, including diffusion-weighted imaging, demonstrated small ischemic lesions consistent with lacunar infarcts without evidence of large territorial infarction or hemorrhagic transformation. This case highlights the diagnostic challenges of posterior circulation stroke and emphasizes that early vascular imaging and timely mechanical thrombectomy can result in favorable outcomes, even in elderly patients with acute BAO.

## Introduction

Basilar artery occlusion (BAO) is an uncommon yet devastating cause of ischemic stroke, accounting for approximately 1% of all ischemic strokes. Because the basilar artery supplies critical structures such as the brainstem, cerebellum, thalamus, and occipital lobes, its occlusion can rapidly lead to severe neurological disability or death if timely reperfusion is not achieved [1].

The clinical presentation of BAO is notably heterogeneous. Symptoms may range from dizziness, vertigo, and nausea to profound altered consciousness, coma, or locked-in syndrome. Distal or “top-of-the-basilar” occlusions frequently involve the midbrain and thalamus and often manifest with altered mental status, oculomotor disturbances, ataxia, and behavioral changes, sometimes in the absence of prominent motor deficits [2]. This variability contributes to frequent diagnostic delays in emergency settings, where posterior circulation strokes remain under-recognized [3].

Historically, BAO has been associated with poor outcomes, with mortality rates approaching 80-90% in untreated patients. Although intravenous thrombolysis has long been used in the management of acute ischemic stroke, recanalization rates in posterior circulation large-vessel occlusions remain limited. Mechanical thrombectomy has been firmly established as the standard of care for anterior circulation large-vessel occlusion. More recently, randomized controlled trials, including Endovascular Treatment for Acute Basilar Artery Occlusion (ATTENTION) and Basilar Artery Occlusion Chinese Endovascular (BAOCHE), have provided growing evidence supporting the use of endovascular therapy (EVT) for BAO in selected patients [4-6].

In this report, we describe a case of acute BAO with a nonspecific clinical presentation that was successfully treated with mechanical thrombectomy, resulting in complete neurological recovery. This case highlights the diagnostic challenges of posterior circulation stroke and underscores the importance of early recognition and timely endovascular intervention.

## Case presentation

An 80-year-old woman with a history of untreated hypertension was brought to the emergency department by ambulance for acute neurological symptoms of less than 24 hours’ duration. She was last known to be well at approximately 3:00 AM, when she awoke independently to use the bathroom without difficulty. After returning to bed, she was later found by family members to have developed sudden-onset vomiting, marked gait instability, inability to stand without assistance, disorientation, somnolence, abnormal eye movements, and impaired coordination. Prior to symptom onset, the patient was fully independent in all activities of daily living. 

On arrival to the emergency department at 9:30 AM, the patient appeared somnolent and hypoactive but was arousable to verbal stimuli. She demonstrated bradylalia and slurred speech and was able to answer simple questions. Orientation was fluctuating; she recognized that she was in a hospital and that she had been transported by ambulance, but was unable to clearly describe her symptoms or the events leading to presentation. Neurological examination was limited by her mental status and poor cooperation. Generalized weakness was noted, although she intermittently demonstrated preserved motor coordination. Cerebellar testing could not be reliably performed. Her Glasgow Coma Scale score was 12 (E3V4M5), and the National Institutes of Health Stroke Scale (NIHSS) score was 14.

**Figure 1:**
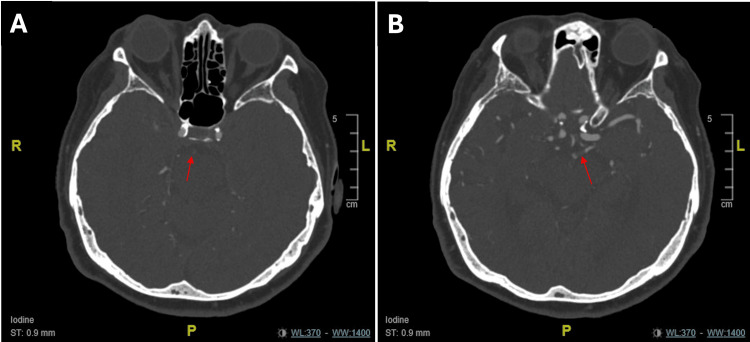
CT angiography of the head (A) Red arrow shows the absence of contrast opacification of the basilar artery, consistent with acute basilar artery occlusion. (B) Red arrow shows the absence of contrast opacification of the P1 segment of the left posterior cerebral artery

**Figure 2:**
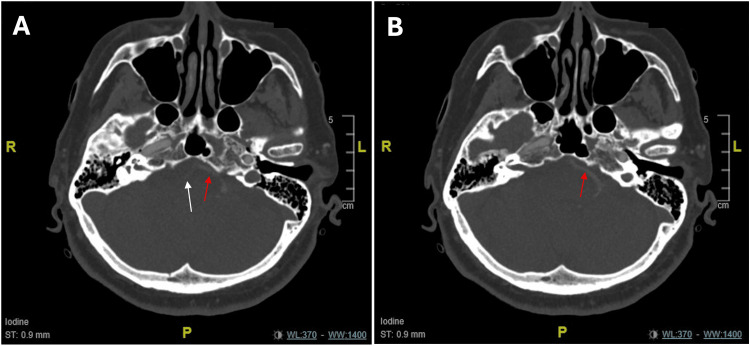
CT angiography of the head (A) The white arrow indicates a hypoplastic right vertebral artery with calcifications. The red arrow indicates the left vertebral artery with reduced contrast opacification compared with the internal carotid arteries and the middle cerebral arteries. (B) The red arrow shows reduced contrast opacification of the left vertebral artery when compared with the internal carotid arteries and middle cerebral arteries

A noncontrast brain CT demonstrated moderate-to-severe chronic small vessel disease without evidence of acute intracranial hemorrhage. CT angiography of the head and neck revealed the absence of contrast opacification of the basilar artery and the P1 segment of the left posterior cerebral artery (Figure 1). The left vertebral artery showed reduced contrast opacification compared with the internal carotid and middle cerebral arteries. The right vertebral artery appeared hypoplastic with calcifications (Figure 2). Posterior communicating arteries were present. No intracranial aneurysms or significant carotid artery stenosis were identified.

**Figure 3:**
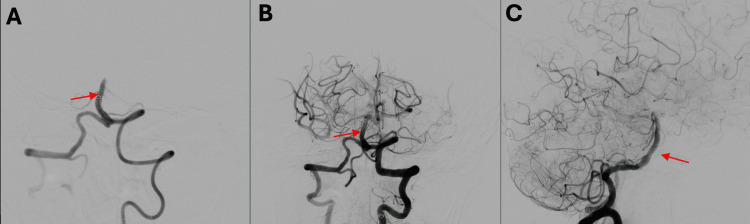
Cerebral angiography via the left vertebral artery during mechanical thrombectomy (A) The red arrow shows the catheter positioned at the level of the basilar artery with resistance to contrast injection, resulting in the absence of contrast filling of the posterior circulation. (B) The red arrow shows the basilar artery after successful recanalization, with free contrast flow into the posterior circulation on coronal view. (C) The red arrow shows the recanalized basilar artery with restored contrast flow into the posterior circulation on sagittal view

Based on the acute clinical presentation and vascular imaging findings, a diagnosis of acute BAO was established. Intravenous thrombolysis was not administered due to the estimated time from symptom onset exceeding the recommended therapeutic window. The patient was immediately transferred to the angiography suite at approximately 11:00 AM, and successful reperfusion was achieved approximately nine hours after she was last known well, where digital subtraction angiography confirmed the occlusion, and mechanical thrombectomy was performed, achieving complete recanalization (Figure 3) (Videos 1-3).

**Video 1:** Cerebral arteriography demonstrating basilar artery occlusion Digital subtraction arteriography via the left vertebral artery shows absence of antegrade contrast flow into the basilar artery, consistent with acute basilar artery occlusion

**Video 2:** Cerebral arteriography showing basilar artery recanalization (sagittal view) Postprocedural digital subtraction arteriography demonstrates the recanalized basilar artery with restored contrast flow into the posterior circulation on sagittal view

**Video 3:** Cerebral arteriography showing basilar artery recanalization (coronal view) Postprocedural digital subtraction arteriography shows successful recanalization of the basilar artery with restored antegrade contrast flow into the posterior circulation on coronal view

The patient showed rapid neurological improvement. Within 24 hours, she had returned to her baseline neurological status. Follow-up brain MRI performed 24 hours after the procedure demonstrated small ischemic lesions consistent with lacunar infarcts in the right frontal subcortical region and left cerebellar hemisphere in the setting of severe chronic small-vessel disease. No large territorial infarctions or hemorrhagic transformation were identified. The patient continued to improve clinically and was discharged home without significant neurological deficits. At the time of discharge, her Glasgow Coma Scale score was 15, and she had fully returned to her baseline functional status, remaining independent in activities of daily living. Neurological examination did not reveal persistent focal deficits. Her functional outcome was excellent, with a modified Rankin Scale (mRS) score of 0.

## Discussion

Posterior circulation strokes account for approximately 15-20% of all ischemic strokes, but they carry a disproportionate burden of disability and mortality. Among these, acute BAO represents one of the most devastating phenotypes and has historically been associated with mortality rates exceeding 80% in the absence of recanalization. Although contemporary randomized trials have demonstrated the benefit of EVT in selected patients, early clinical recognition of BAO remains a major barrier to timely reperfusion [1].

The present case illustrates the diagnostic complexity of BAO presenting without prominent focal motor deficits. Symptoms such as vertigo, vomiting, gait instability, fluctuating mental status, and oculomotor abnormalities reflect involvement of the brainstem reticular activating system, cerebellar pathways, and midbrain structures supplied by the basilar apex and its perforators. In such scenarios, the absence of dense hemiparesis may reduce clinical suspicion for large-vessel occlusion. This phenotypic heterogeneity contributes substantially to diagnostic delay.

In emergency department settings, this case highlights the potential value of early vascular imaging in selected patients presenting with acute vestibular syndrome accompanied by altered mental status or ocular motor abnormalities. Rather than representing a universal recommendation, this observation may serve as a practical diagnostic consideration to improve early recognition of posterior circulation stroke [7-8].

This case also highlights the limitations of the NIHSS in posterior circulation stroke. Although the patient’s NIHSS score was 14, much of the deficit was attributable to an altered level of consciousness and cerebellar dysfunction rather than hemispheric motor findings. The NIHSS, originally developed for anterior circulation events, may therefore underestimate or incompletely characterize the severity of brainstem ischemia. Recognition of this limitation is particularly important in emergency settings where treatment decisions may be influenced by severity thresholds.

Recent randomized trials have reshaped the therapeutic landscape of BAO. The ATTENTION trial demonstrated that EVT significantly improves functional outcomes and reduces mortality compared with best medical therapy in patients treated within 12 hours of symptom onset [9]. The BAOCHE trial extended this benefit to carefully selected patients treated between six and 24 hours, while the BEST trial, although neutral in its intention-to-treat analysis, was limited by substantial crossover and insufficient statistical power [10-11]. Collectively, these studies support EVT as an important treatment option in appropriately selected patients with BAO. Importantly, these trials consistently demonstrate that successful reperfusion is the strongest determinant of a favorable outcome.

The favorable recovery observed in this patient likely reflects several recognized prognostic factors. She had preserved premorbid functional status (mRS: 0), moderate baseline neurological severity, and achieved complete reperfusion. Although advanced age has traditionally been associated with poorer outcomes, emerging evidence suggests that chronological age alone should not automatically preclude consideration of EVT, particularly when favorable imaging and clinical characteristics are present.

Nevertheless, outcomes in BAO remain highly variable, and individual prognosis depends on multiple factors, including collateral circulation, infarct burden, baseline neurological status, and time to reperfusion. This case, therefore, should be interpreted as an illustration of a possible favorable outcome rather than a broadly generalizable expectation.

In summary, this case highlights several clinically relevant teaching points. First, BAO may present without dense motor deficits and should be considered in elderly patients presenting with acute vestibular syndrome accompanied by altered mental status or ocular motor abnormalities. Second, the NIHSS has important limitations in posterior circulation stroke. Third, timely EVT can result in favorable neurological outcomes in selected patients, even in advanced age, when appropriate clinical and imaging conditions are present.

## Conclusions

BAO is a rare but devastating cause of ischemic stroke that frequently presents with nonspecific neurological symptoms, contributing to delays in diagnosis and treatment. A high index of suspicion for posterior circulation stroke is essential in patients presenting with acute vertigo, ataxia, altered mental status, or oculomotor abnormalities. Early vascular imaging and timely mechanical thrombectomy can result in excellent functional outcomes, even in elderly patients, when appropriate patient selection is applied.
